# Effect of water quality on causes of calf mortality in cattle-farm-associated epidemics

**DOI:** 10.5194/aab-67-25-2024

**Published:** 2024-01-16

**Authors:** Mohammed A. Kamal, Mahmoud A. Khalf, Zakia A. M. Ahmed, Jakeen A. Eljakee, Rashed A. Alhotan, Mohammed A. A. Al-Badwi, Elsayed O. Hussein, Branislav Galik, Ahmed A. Saleh

**Affiliations:** 1 Department of Veterinary Hygiene and Management, Faculty of Veterinary Medicine, Cairo University, 11221, Giza, Egypt; 2 Department of Microbiology, Faculty of Veterinary Medicine, Cairo University, 11221, Giza, Egypt; 3 Department of Animal Production, College of Food & Agriculture Sciences, King Saud University, P.O. Box 2460, Riyadh 11451, Saudi Arabia; 4 Institute of Nutrition and Genomics, Slovak University of Agriculture in Nitra, Slovakia; 5 Department of Poultry Production, Faculty of Agriculture, Kafrelsheikh University, Kafr El-Sheikh, 333516, Egypt

## Abstract

Poor-quality drinking water plays a detrimental role in the suppression of calf immunity, giving rise to an increased rate of calf mortality. The present study aims to evaluate the causes of calf mortality in beef and dairy farms in relation to drinking water quality (DWQ). A convenience sample of 132 Egyptian cattle farms suffering from emerging epidemics was surveyed by collecting drinking water samples for physicochemical and microbial analysis and using a questionnaire to record hygienic risk factors affecting calf health. Statistical analysis correlates water parameters with rates of calf diarrhea, respiratory problems, severe depression, sudden death and mortality. High percentages of water sample quality parameters, e.g. pH, total dissolved solids (TDSs), hardness, chloride, nitrate, sulfate, total colony count (TCC) and total coliform count (TCFC), are above permissible limits. Water parameters, except pH, show a significant moderate positive correlation with causes of calf mortality ( ρ 0.331–0.66) in winter and summer. Each cause of calf mortality was predicted by a specific water parameter, and the water nitrate level was the highest predictor, with the highest values ( β = 0.504–0.577), followed by the water TDS, sulfate and microbial levels. Weak to moderate correlation ( ρ 0.151–0.367) was found between calf mortality causes and some hygienic risk factors such as operation type, calf housing, calf feeders, bedding type, water source, water pipe type, drinker lining and wheel dipping. We could conclude that DWQ greatly affects causes of calf mortality, but we cannot exclude some farm hygienic risk factors.

## Highlights

- Seventy to 97 % of the total water needed by calves was from drinking water.
- In addition to water quantity needs in calves, drinking water quality is important for calf health and productivity.
- Water quality depends on its source and contamination from abiotic and biotic factors, either dissolved nutrients or directly from urine or fecal matter.
- There is an association between different causes of calf mortality rates and the presence of drinking water contaminants that cause severe health and performance problems.

## 1 Introduction

Water, as an essential nutrient, is second only to oxygen for sustaining life and maximizing the growth and performance of cattle calves. The water requirements per unit of bovine body mass are higher than those of any other mammal (Beede, 2012).

Drinking water accounted for 70 %–97 % of the total amount of water needed by calves. Drinking water quality (DWQ) is crucial for the health and productivity of calves, in addition to water quantity. The source and level of biotic and abiotic contamination – whether from dissolved nutrients or directly from feces or urine – determine the quality of the water. While surface runoff or subterranean water carrying labile levels of dissolved nutrients is the primary source of charge for ponds and dugouts, water from deep underground wells may have a significant mineral content if it originates from marine shales (Kamal et al., 2019).

Calf DWQ is mainly evaluated by major parameters, of which physicochemical parameters, e.g. including pH, total dissolved solids (TDSs), hardness, excessive amounts of minerals (such as nitrates, chloride and sulfates) and microbial loads, e.g. total colony count (TCC) and total coliform count (TCFC) in the water, are the most detrimental agents reducing DWQ (Willms et al., 2002).

These DWQ parameters, by variable mechanisms, affect calf health, immunity, morbidity and various causes of calf mortality in cattle herds (Alves et al., 2017). Seasonal climatic factors and farm hygienic risk factors such as operation type, hygienic standards, housing factors and water distribution systems are among the factors that have an impact on calf mortality (Makris et al., 2014).

The aim of the study is to survey some dairy and beef farms that have emerging epidemics in Egypt, to assess the quality and health aspects of water intended for calf consumption and to determine whether there is an association between different causes of calf mortality rates and the presence of drinking water contaminants that cause severe health and performance problems.

## 2 Materials and methods

### 2.1 Field survey

**Figure 1:**
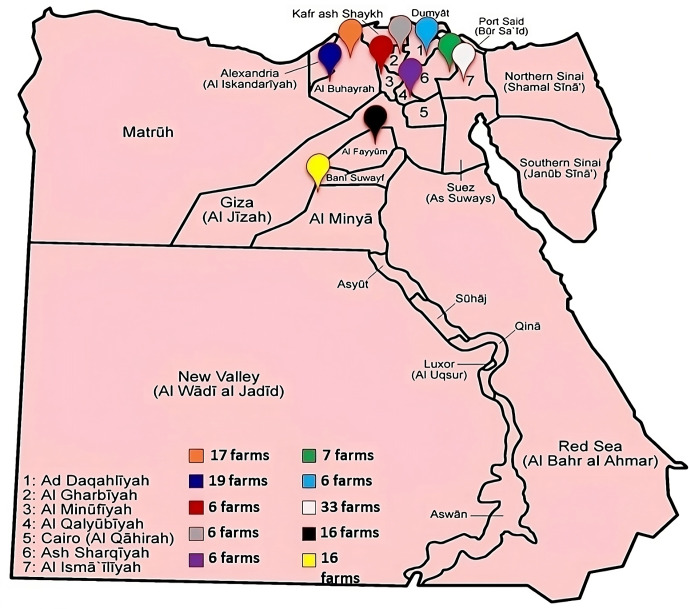
Count and locations of the cattle farms included in the study survey.

*Study area and period.* A field study was conducted during the period from October 2016 to September 2018 in four districts around Egypt: the West Delta (including Behira and the Alex Desert Road), the Middle Delta (including Menoufia and Gharbia), the East Delta (including Kaluobia, Sharkia, Dakahlia and the Ismailia Desert Road) and Upper Egypt (including Fayoum, Beni-Suef and Minya). Representative water samples were collected from water troughs in calf houses at beef cattle farms ( n=60 ), dairy cattle farms ( n=60 ) and dairy beef mixed farms ( n=12 ), for a total of 132 farms in the investigated area (Fig. 1).

*Study design.* The protocol of the study involved steps that aim to investigate the hygienic DWQ in calf houses on both beef and dairy cattle farms located in different areas in Egypt and suffering from emerging epidemics. For this purpose, water samples were collected from calf houses for physicochemical examination and indicator microbe enumeration. Selection criteria were based on the previous history of calf health problems and emerging epidemics associated with drinking water in the investigated areas (Fig. 1). A structured questionnaire was assembled to identify the associated hygienic risk factors on each farm. The obtained data were analysed to identify the risk associated with the occurrence and spread of calf mortality on these farms (Table 2).

*Questionnaire survey.* A structured questionnaire was prepared, including full farm identification and information regarding prevalence and risk indicators of calf mortalities in dairy and beef herds including those attributed to both calves and farms (Table 2). Household attributes include housing type, contact with other animals, waste handling, carcass disposal methods and bedding type; water attributes include water source type, tank type, pipe type and drinker lining; and disinfection attributes include wheel dip, foot dip, hoof dip and teat dip. Finally, data related to recorded causes of calf mortality include scours or diarrhea rate, respiratory problem percentage, severe depression or off-feed rate, sudden death and mortality rate according to USDA APHIS (2009). All data were obtained from clinical records of the farm and interviews with the workers, owners and veterinarians.

### 2.2 Water sampling

A total of 132 water samples comprised three sources: underground water, surface water and commercial tap water. Samples were collected from 132 calf houses on dairy, beef and mixed cattle farms in different governorates in Egypt. Water samples were collected equally in the winter (December, January, February) and summer (June, July, August) seasons from all the farms under investigation.

For the physicochemical evaluation, 1 L plastic bottles with screw caps, which were clean and dry, were used to collect water samples. Conversely, for microbiological analysis, 1 L sterilized glass bottles with screw caps were employed. These glass containers were sterilized in a hot-air oven at 170 ${}^{\circ }$ C for 60 min and rinsed thoroughly with the respective water to be sampled before collection. All the samples gathered were stored at 4 ${}^{\circ }$ C and analysed within the subsequent 48 h (Kamal et al., 2023).

At the same time, direct sampling of water was performed using a type of dip slide (©Liofilchem^®^): a CONTACT SLIDE CHROM 2 (ChromaticTM Coli Coliform/Plate Count Agar + TTC + Neutralizing) flex dip slide with a chromogenic selective medium for detection and enumeration of *E. coli* together with coliforms and a non-selective medium for the total bacterial count. Then, they were used according to the ISO (2004).

Each sample was labelled and identified, showing its source, site, type of watering system and date of sampling. All the collected samples were transferred to the laboratory within 2 h.

### 2.3 Laboratory examination of water samples

*Chemical examination.* All chemical examinations of collected water samples were performed in the laboratory of the Department of Veterinary Hygiene and Management, Faculty of Veterinary Medicine, Cairo University, as recommended by Rice et al. (2012). Temperature was measured at the time of sampling by means of an ordinary thermometer (range 0–100 ${}^{\circ }$ C). The pH values of the water samples were determined by an electrometric pH meter (pHep^®^ HI 98107 – Italy). TDSs were measured by using a waterproof EC–TDS–NaCl (% / ${}^{\circ }$ C) meter (HI 9835 – Italy). Total hardness was estimated by using the “EDTA titrimetric method”. Chlorides (Cl ${}^{-}$ ) were estimated by the argentometric method. Nitrate (NO ${}_{3}^{-}$ ) was estimated by the ultraviolet spectrophotometric screening method. Sulfates (SO ${}_{4}^{2-}$ ) were determined by the gravimetric methods with drying of residues.

*Microbiological examination of water samples.* We used the pour plate method to enumerate the TCC. Furthermore, we used the multiple tube fermentation technique to measure the TCFC, in compliance with the instructions provided by Rice et al. (2012).

*Dip slides.* As directed by the manufacturer's handbook and technical sheet as well as the guidelines supplied by it, the incubation and assessment of dip slides were completed (ISO, 2003).

### 2.4 Statistical and data analysis

For analysis of data, the Statistical Package for Social Sciences software version 25.0 (SPSS Inc., Chicago, IL) was used. Initially, all information gathered via a questionnaire was coded into variables. The normality of the data was tested using the Kolmogorov–Smirnov test. Both descriptive and inferential statistics involving the Wilcoxon signed rank test, Kruskal–Wallis H test and linear regression were used to present the results. Effect size was calculated by Cohen's d and the $\eta^{2}$ value. For each test, p<0.05 was considered statistically significant according to Campbell (2021).

## 3 Results

The survey applied to 46 farms in the West Delta (17 in Behira and 19 in the Alex Desert Road), 12 farms in the Middle Delta (6 in Menoufia and 6 in Gharbia), 52 farms in the East Delta (6 in Kaluobia, 7 in Sharkia, 6 in Dakahlia and 33 in the Ismailia Desert Road) and 22 farms in Upper Egypt (16 in Fayoum and 6 in Beni-Suef and Minya) (Fig. 1).

### 3.1 Cattle operations included in the survey

Overall, 2.3 % of the cattle operations were classified as small ( <100 head), 40.9 % were medium (100 to 500 head) and 56.8 % were large ( >500 head). Although these operations were a convenience sample of cattle farms across the Egyptian country, they reflect the diversity of operations and herd sizes present in the cattle population (Table 1).

**Table 1:** Count ( N ) and percentage (%) of the survey farms in each herd size class.

Operation type	N (%) according to herd size ${}^{*}$			Total
	Small	Medium	Large	
Dairy	1 (0.8)	27 (20.5)	32 (24.2)	60 (45.5)
Beef	1 (0.8)	25 (18.9)	34 (25.8)	60 (45.5)
Mixed	1 (0.8)	2 (1.5)	9 (6.8)	12 (9.1)
Total	3 (2.3)	54 (40.9)	75 (56.8)	132

${}^{*}$ Small, <100 head; medium, 100–500 head; large, >500 head.

### 3.2 The questionnaire surveys

The Holstein–Friesian (HF) breed was present in 94.4 % of the survey farms, according to descriptive data for each questionnaire item (Table 2). In terms of calf housing, 75 % of the farms surveyed utilized individual hutches. A total of 96.2 % of the farms employed manual troughs for calf drinking, whereas 68.9 % of the farms used underground water as their drinking water source.

Causes of calf mortality were recorded in both the winter (W) and summer (S) seasons. The results revealed higher quartiles in winter than in summer, except for sudden death rates (Table 3).

**Table 2:** Count ( N ) and percentage (%) of the farms in the different questionnaire-recorded items and risk factor profiles during the study survey.

Variable	N (%)	Variable	N (%)	Variable	N (%)
Farm record type		Cooling system		Water pipe type	
– Computerized	87 (65.9)	– No cooling	59 (44.7)	– Metal	67 (50.8)
– Handwritten	45 (34.1)	– Sprinkler	55 (41.7)	– Plastic	65 (49.2)
Animal ID type		– Foggers	17 (12.9)	Water tank type	
– Electronic ID	23 (17.4)	– Cooling pads	1 (0.8)	– Concrete	60 (45.5)
– Collars	14 (10.6)	Ventilation type		– Fibre glass	21 (15.9)
– Ear tag	93 (70.5)	– Open	107 (81.1)	– Galvanized steel	45 (34.1)
– Branding	2 (1.5)	– Closed	25 (18.9)	– Plastic	6 (4.5)
Cattle breeds		Waste handling		Calf feeder type	
– Holstein–Friesian	136 (94.4)	– Composting	32 (24.2)	– Bucket	78 (59.1)
– Simmental	3 (2.1)	– Picket dam	47 (35.6)	– Trough	49 (37.1)
– Brown Swiss	1 (0.7)	– Left on pasture	38 (28.8)	– Manger	5 (3.8)
– Crossbreed	3 (2.1)	– Landfill	13 (9.8)	Calf feeder material	
– Baladi	1 (0.7)	– Alley scraper	1 (0.8)	– Galvanized steel	62 (47)
Calf housing type		– Manure pack	1 (0.8)	– Cement	50 (37.9)
– Individual hutch	54 (75)	Carcass disposal		– Wooden	3 (2.3)
– Collective hutch	12 (16)	– Buried	41 (31.1)	– Plastic	15 (11.4)
– Catch stall	5 (31.3)	– Landfill	51 (38.6)	– Stainless steel	2 (1.5)
– Stock or tied-up stall	1 (3.2)	– Rendered	1 (0.8)	Wheel dip disinfectant	
Bedding type		– Composted	1 (0.8)	– Absent	69 (52.3)
– Sand	87 (65.9)	– Incinerated	13 (9.8)	– Phenol	43 (32.6)
– Soil	1 (0.8)	– Burned	25 (18.9)	– Formalin	20 (15.2)
– Straw	41 (31.1)	Water source		Disinfectant change	
– Artificial mats	3 (2.3)	– Underground	91 (68.9)	– Weekly	56 (88.9)
Physical contact		– Tap	32 (24.2)	– Monthly	7 (11.1)
– No contact	93 (70.5)	– Surface	9 (6.8)	Foot dip disinfectant	
– Sheep	19 (14.4)	Drinker type		– Absent	102 (77.3)
– Beef	9 (6.8)	– Troughs	127 (96.2)	– Phenol	26 (19.7)
– Buffalo	6 (4.5)	– Automatic cups	5 (3.8)	– Formalin	4 (3)
– Goat	5 (3.8)	Drinker lining		Disinfectant change	
– Donkey	4 (3)	– Ceramic	29 (22)	– Daily	20 (66.7)
– Dog	2 (1.5)	– Cement	92 (69.7)	– Weekly	10 (33.3)
– Horse	1 (0.8)	– Stainless steel	5 (3.8)		
– Poultry	2 (1.5)	– Galvanized steel	2 (1.5)		
– Camel	1 (0.8)	– Aluminium	3 (2.3)		
		– Plastic	1 (0.8)		

**Table 3:** Three frequency quartiles (Q1, Q2 (median), Q3) of the causes of calf mortality in the survey farms in winter (W) and summer (S).

Percentiles ${}^{*}$	Diarrhea		Respiratory		Severe		Sudden death		Mortality	
			problems		depression		rate		rate	
	W	S	W	S	W	S	W	S	W	S
Q1	4.6	3.4	1.3	0.8	0.1	0.1	0	0.125	2.6	1.9
Q2	5	3.8	1.7	1.25	0.4	0.4	0.2	0.3	3.25	2.7
Q3	6.975	5	3.875	2.975	1.575	1.1	0.7	0.7	5.475	4.05

${}^{*}$ Percentiles equal the frequency quartiles (quartiles are the alternative to the arithmetic mean in non-normally distributed data) and Q2 is the median.

### 3.3 Laboratory analysis of the water samples

Laboratory analysis of the collected water samples from calf drinkers of the survey farms revealed various results for both chemical and microbial analyses. Physicochemical parameters showed high variation between Q1 and Q3. Microbiological parameters recorded higher levels in summer than in winter (Table 4).

Spearman rank correlation analysis showed statistically significant positive correlations ( p<0.05 ). Specifically, water TDS levels exhibited a strong positive correlation with parameters such as hardness ( ρ = 0.77), chloride ( ρ = 0.89), nitrate ( ρ = 0.32) and sulfate ( ρ = 0.78). Furthermore, a significant positive correlation ( p < 0.05) was observed between water TCC and TCFC, with a correlation coefficient ( ρ ) of 0.84.

**Table 4:** Frequency of the three quartiles (Q1, Q2 (median), Q3) of the water physicochemical and microbial W and S quality parameters on the survey farms.

Percentiles	pH	TDS	TH	Cl ${}^{-}$	NO ${}_{3}^{-}$	SO ${}_{4}^{2-}$	TCC (W)	TCC (S)	TCFC (W)	TCFC (S)
Q1	8.1	305	285	150	2	66	4.1 × 10 ${}^{4}$	6.95 × 10 ${}^{4}$	4.4 × 10 ${}^{3}$	6.7 × 10 ${}^{3}$
Q2	8.4	680	472	240	4	100	32 × 10 ${}^{5}$	59 × 10 ${}^{5}$	2.6 × 10 ${}^{5}$	5.1 × 10 ${}^{5}$
Q3	8.8	1472.5	698	448	8	141.5	42.8 × 10 ${}^{6}$	74 × 10 ${}^{6}$	5.3 × 10 ${}^{5}$	9.4 × 10 ${}^{5}$

TH: total hardness; Cl ${}^{-}$ : chloride; NO ${}_{3}^{-}$ : nitrate; SO ${}_{4}^{2-}$ : sulfate; TCC: total colony count; TCFC: total coliform count.

### 3.4 Evaluation of the seasonal effect

To evaluate the potential seasonal effects and significant differences between the results obtained during winter and summer, we conducted inferential statistics using the Wilcoxon signed rank test. The results indicated a significant difference between the winter and summer in terms of microbial water parameters, specifically TCC and TCFC. The mean ranks were 65.5 and 61.5, and the Z values were 9.89 and 9.59, respectively. Furthermore, Cohen's d values for these parameters were 0.86 and 0.83, indicating a significant difference between the two seasons. Additionally, the test revealed a significant difference between the rates of calf mortality causes in winter and summer, as summarized in Table 5.

**Table 5:** Wilcoxon signed mean ranks with farm numbers ( N ), Ties number, Z value and effect size (RdR) for differences between W and S rates of calf mortality causes.

Variable	+ mean rank ( N ) ${}^{*}$	- mean rank ( N ) ${}^{*}$	Ties ${}^{*}$	$Z^{*}$	Cohen's $d^{*}$
Diarrhea (W) – diarrhea (S)	66.5 (132)	0	0	10.007	0.87
Respiratory (W) – respiratory (S)	66.5 (132)	0	0	10.026	0.87
Depression (W) – depression (S)	50.7 (40)	17.0 (31)	61	4.372	0.38
Sudden death (W) – sudden death (S)	58.7 (23)	47.4 (76)	33	4.155	0.36
Mortality (W) – mortality (S)	66.5 (132)	0	0	10.01	0.87

${}^{*}$ Positive + mean rank: this denotes the level of samples at which the first variable is larger than the second one. N : this denotes the number of samples in each variable. Negative - mean rank: this denotes the level of samples at which the first variable is smaller than the second one. Ties: this denotes the number of samples with equal results. Z : this denotes the level of the difference between two related variables (i.e. ambient and water temperature), and if the Z value equals zero, there is no difference. Cohen's d value denotes the measurement of the effect of the first variable on the results of the second related variable.

### 3.5 Inferential statistics

**Figure 2:**
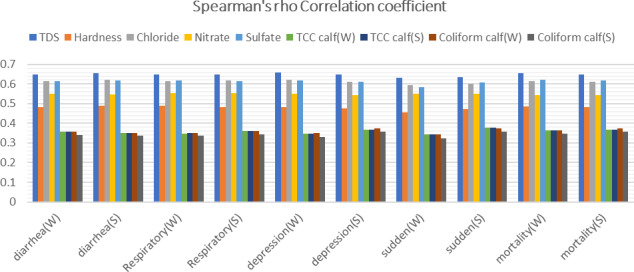
Spearman's ρ correlation coefficient between water quality parameters with causes of calf mortality in both winter (W) and summer (S).

The results indicated a statistically significant correlation ( p<0.05 ) between all the examined DWQ parameters and causes of calf mortality, including rates of diarrhea, respiratory problems, severe depression, sudden death and overall mortality rate, as depicted in Fig. 2. Notably, the highest correlation values were associated with water parameters such as TDS, chloride and sulfate, while microbial counts exhibited relatively lower correlation values. The only exception to this correlation trend was observed with pH.

**Figure 3:**
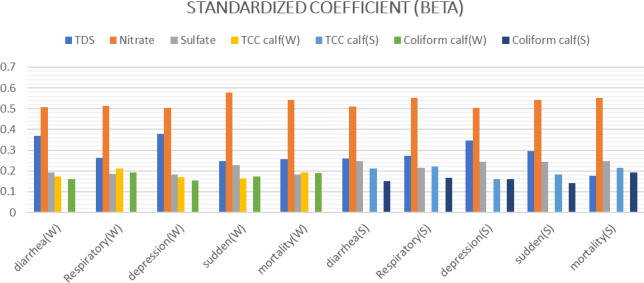
Linear regression standardized coefficient ( β ) of the best predictor water quality parameters which significantly correlated with different causes of calf mortality in W and S.

Linear regression analysis demonstrated that water parameters such as TDS, nitrate, sulfate, TCC in summer and TCFC in both summer and winter served as significant predictors of the causes of calf mortality. The $R^{2}$ values ranged from 0.541 to 0.607, indicating the extent to which these parameters could explain the variance in calf mortality (Fig. 3).

**Figure 4:**
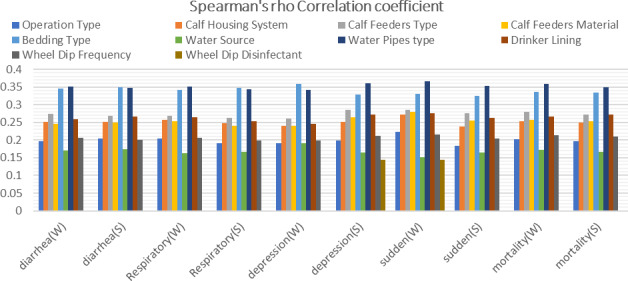
Spearman's ρ correlation coefficient between the significant risk factors with causes of calf mortality in both W and S.

Spearman rank correlation revealed a statistically significant correlation ( p<0.05 ) between specific farm risk factors, such as operation type, calf housing system, calf feeder type, calf feeder material, bedding type, water source, water pipe type, drinker lining, wheel dip frequency and wheel dip disinfectant as well as causes of calf mortality in both winter and summer (Fig. 4).

To evaluate of the effect of each farm risk factor type on different causes of calf mortality, inferential statistics using the Kruskal–Wallis H test to obtain mean ranks and the Kruskal–Wallis H value and to calculate the effect size by $\eta^{2}$ measures of association were performed. The test shows that the operation type affects causes of calf mortality, with mean ranks of 76.7, 72.6 and 55 for beef, mixed and dairy, respectively, with an average $\eta^{2}$ value of approximately 0.1.

The calf housing system affects causes of calf mortality, with mean ranks of 65, 59.9, 56.3 and 52.8 for collective hutch, catch stall, individual hutch and stock or tied-up stall, respectively, with an average $\eta^{2}$ value of approximately 0.06.

The calf feeder type affects causes of calf mortality, with mean ranks of 100.6, 77.8 and 57.2 for manger, trough and bucket, respectively, and an average $\eta^{2}$ value of approximately 0.07. The calf feeder's material affects the causes of calf mortality, with mean ranks of 81.1, 80.6, 60, 56.3 and 45.8 for cement, wooden, plastic, galvanized steel and stainless steel, respectively, with an average $\eta^{2}$ value of approximately 0.07.

The bedding type affects calf mortality, with mean ranks of 76.9, 67.8, 45.3 and 29.1 for sand, straw, soil and artificial mats, respectively, with an average $\eta^{2}$ value of approximately 0.06.

The water source affects calf mortality, with mean ranks of 109, 72.6 and 37.3 for the surface, underground and tap water, respectively, and an average $\eta^{2}$ value of approximately 0.18. The water pipe type affects the causes of calf mortality, with mean ranks of 80.1 and 53.3 for plastic and metal, respectively, and an average $\eta^{2}$ value of approximately 0.11. The drinker lining type affects causes of calf mortality with mean ranks of 73.6, 57.4, 61.2, 49.1, 45.4 and 52.8 for cement, galvanized steel, aluminium, ceramic, stainless steel and plastic, respectively, and an average $\eta^{2}$ value of approximately 0.12.

The wheel dip disinfectant affects only the depression rate in summer and the sudden death rate in winter, with mean ranks of 78.8, 72.6 and 57.5 for monthly change, no change and weekly change, respectively, and an average $\eta^{2}$ value of approximately 0.05. The wheel dip change frequency affects causes of calf mortality, with mean ranks of 73, 66.5 and 56.2 for no disinfectant use, formalin and phenol, respectively, and an average $\eta^{2}$ value of approximately 0.07.

## 4 Discussion

Drinking water is considered an important nutrient for livestock calf health and production but is prone to different quantity and quality continuous issues. Due to either seasonal and climatic changes, different water sources from a dugout, ponds or tap water or chemical and microbial contamination by many factors, all these water problems affect both the health and performance of cattle calves (Kamal et al., 2019). The present study has focused on determining whether there is an association between causes of calf mortality and the presence of DWQ issues that cause severe health and performance problems.

The study results indicate that a large number of the survey farms showed levels of pH, TDS, hardness, chloride, nitrate, sulfate, TCC and TCFC of the permissible limits according to the CCME (1999) guidelines for livestock as shown in Table 4.

The study survey recorded causes of calf mortality in each farm with variable rates of diarrhea, respiratory problems, severe depression, sudden death and mortality around the year, as shown in Table 3. Statistical analysis showed a moderate positive correlation ( ρ 0.331–0.66) between the causes of calf mortality and all water parameters except pH in both the winter and summer seasons (Fig. 3). However, with linear regression, we found that each cause of calf mortality has specific water parameters that predict its value better, as shown in Fig. 4.

Nitrate is the highest predictor of all causes of calf mortality, with the highest β (0.504–0.577), as was previously reported, where the water nitrate level greatly affects dairy and calf health indicators (Dahal et al., 2020; Sharifi et al., 2022; Wagner and Engle, 2021). Nitrate can be reduced to nitrite in the rumen by bacteria. For this reason, ruminant livestock is more susceptible to nitrate poisoning than mono-gastric animals (Mutwedu et al., 2020; Sanders and Langeveld, 2020), and nitrate is also implicated in poor growth, infertility problems, vitamin A deficiencies (Ibrahim, 2015; El Mahdy et al., 2016), impaired thyroid function as well as immune function and growth rate (Serrano-Nascimento and Nunes, 2022; Arshad et al., 2021).

Then, followed by TDS with β (0.176–0.38), as previously reported, the water TDS level is a pre-indicator of poor DWQ, and its level is greatly influenced by other water parameters such as chloride and sulfate levels (Memon et al., 2023). High levels of TDS decrease feed intake, water intake, growth and production, as mentioned by Patra et al. (2023). In contrast, some authors have declared that high water TDS levels may not be such a problem and may not affect calf health and production (Phillips et al., 2015; Olkowski, 2009).

Sulfate with β (0.183–0.214), which is reported to have a laxative effect, synergizes with molybdenum and causes deficiency of essential minerals such as Cu, Se and Fe as well as vitamins such as thiamin and vitamin E. Sulfate has a negative effect on water or feed intake, growth, reproductive organs, muscular structure and brain lesions; affects both humoral and cellular immunity; and increases the rate of infection, morbidity and mortality rate (Beede, 2012; Grout et al., 2006; Patterson et al., 2003; McKenzie et al., 2009; Olkowski et al., 1993; Arthington, 2006). No adverse effects of high water sulfate and the presence of ruminal adaptation for sulfate were reported by Digesti and Weeth (1976) and Weeth and Capps (1972).

For water microbial analysis, TCC with β (0.16–0.2) is a predictor higher than TCFC with β (0.1–0.19) in both the winter and summer seasons. These results are in accordance with Brew et al. (2008), El Emam and El Jalii (2010), Mohammed (2016), Yamahara et al. (2009) and Descheemaeker et al. (2010), who mentioned that good hygienic clean water is needed for cattle calves because a high microbial count in water affects palatability, water intake, production, performance and immunity, causing health problems and disease transmission and slowing calf growth. In contrast, Willms et al. (2002) and Crawford et al. (1997) found that contamination and a high microbial count in water have no adverse effect on cattle.

Statistical correlation revealed that water TDS values significantly correlated with other water chemical parameters: a strong positive correlation with chloride ( ρ = 0.89), sulfate ( ρ = 0.78) and hardness ( ρ = 0.77) and a moderate correlation with nitrate ( ρ = 0.32) (Patra et al., 2023; Memon et al., 2023). Additionally, TCC has a strong positive correlation with TCFC ( ρ = 0.84) (Rusin et al., 1997; Synder et al., 1995). A positive correlation indicates an increase in one parameter followed by an increase in the other with a percent equal to the ρ value.

Seasonal differences in water microbial analysis are evaluated by Cohen's d value, which reveals that there is a significant difference between TCC and TCFC in winter and summer, with positive mean ranks of 65.5 and 61.5, Z values of 9.89 and 9.59 and Cohen's ( d ) values of 0.86 and 0.83 for TCC and TCFC, respectively, which indicate that summer results are higher (Sanchez et al., 1994). Additionally, a seasonally significant difference appears in the causes of calf mortality, with an effect size ( d ) value of 0.87 and a positive mean rank of 36.5, which indicates that summer rates are higher (Arias and Mader, 2011; Reymond et al., 2018; Schütz, 2012; Haan et al., 2010; Morris et al., 2010; Avendaño-Reyes et al., 2010; Farooq et al., 2010; Hammoud et al., 2010).

The difference in the causes of calf mortality between winter and summer is evaluated by Cohen's d value (Table 5), which reveals that winter results are higher than summer results of diarrhea, respiratory problems, severe depression, sudden death and mortality rates in 100 %, 100 %, 30.3 % and 100 % of farms, respectively, with a large effect size ( d = 0.87) for diarrhea, respiratory problems and mortality rate and a small effect size ( d = 0.38) for severe depression, meaning that seasonal change has a strong effect on calf diarrhea, respiratory problems and mortality rates, which tend to increase in winter more than summer, but season has a small effect on the changing rate of severe depression. However, 57.6 % of farms show summer results higher than winter results of sudden death rates with a small effect size ( d = 0.36), meaning that seasonal change has a small effect on sudden calf death rates (Arias and Mader, 2011; Solomon et al., 2010; Reymond et al., 2018; Schütz et al., 2010; Saleh et al., 2023a).

To exclude other risk factors that were recorded through the study questionnaire on each farm (Table 2), we statistically analysed and correlated the factors with the causes of calf mortality (Fig. 4). Weak to moderate correlation ( ρ 0.151–0.367) was found between different causes of calf mortality and some significant risk factors, such as operation type ( ρ 0.183–0.224) (Galal, 2007), calf housing system ( ρ 0.239–0.272) (Samer, 2011; Galal, 2007), calf feeder type ( ρ 0.261–0.285) (USDA APHIS, 2009), calf feeder material ( ρ 0.24–0.279) (Samer, 2011; USDA APHIS, 2009), bedding type ( ρ 0.325–0.358) (Zdanowicz et al., 2004; CCME, 1999; Saleh et al., 2023b), water source ( ρ 0.151–0.192) (Melegy et al., 2014; Olson et al., 1997; Emtiazi et al., 2004), water pipe type ( ρ 0.342–0.367) (Juhna et al., 2007; Berry et al., 2006; Niquette et al., 2000), drinker lining ( ρ 0.245–0.275) (September et al., 2007; Lee et al., 2006) and wheel dip frequency ( ρ 0.198–0.215) (Fuqua, 2010; USDA, 2007). Additionally, a weak correlation ( ρ 0.145) was found between the depression rate in summer and the sudden death rate in winter with the significant risk factor wheel dip disinfectant ( ρ 0.145) (WHO, 2008; Fuqua, 2010). Then, by evaluating mean ranks and $\eta^{2}$ values, we can identify the effect size of each risk factor and order individual types as shown above in the Results section.

## 5 Conclusions

The mortality rates of calves are significantly influenced by the quality of drinking water, encompassing both physicochemical and microbial aspects. Notably, there exists a substantial correlation among various physicochemical parameters of the water, including total dissolved solids (TDSs), hardness, chloride, sulfate and nitrate levels. Additionally, a significant correlation is observed between the total colony count (TCC) and the total coliform count per 100 mL (TCFC). Furthermore, seasonal disparities are evident in water microbial counts and in the occurrences of various causes of calf mortality. It is essential to acknowledge that numerous risk factors and hygiene-related standards play a pivotal role in influencing the causes of calf mortality along with the diversity of farm-specific indicators.

Additional research is warranted to delve deeper into the impact of water physicochemical quality on calf growth rates and their future productivity. Additionally, there is a need for more comprehensive studies on waterborne microbes, including the emergence of antibiotic-resistant strains and the formation of biofilms.

## Data Availability

The data presented in this study are available from the corresponding authors upon reasonable request.
